# Detecting PCOS susceptibility loci from genome-wide association studies via iterative trend correlation based feature screening

**DOI:** 10.1186/s12859-020-3492-z

**Published:** 2020-05-04

**Authors:** Xiaotian Dai, Guifang Fu, Randall Reese

**Affiliations:** 1 Department of Mathematical Sciences, SUNY Binghamton University, New York, USA; 2 Idaho National Laboratory, Idaho, USA

**Keywords:** Feature screening, Ultrahigh dimensionality, Sure screening consistency, Categorical data analysis, GWAS

## Abstract

**Background:**

Feature screening plays a critical role in handling ultrahigh dimensional data analyses when the number of features exponentially exceeds the number of observations. It is increasingly common in biomedical research to have case-control (binary) response and an extremely large-scale categorical features. However, the approach considering such data types is limited in extant literature. In this article, we propose a new feature screening approach based on the iterative trend correlation (ITC-SIS, for short) to detect important susceptibility loci that are associated with the polycystic ovary syndrome (PCOS) affection status by screening 731,442 SNP features that were collected from the genome-wide association studies.

**Results:**

We prove that the trend correlation based screening approach satisfies the theoretical strong screening consistency property under a set of reasonable conditions, which provides an appealing theoretical support for its outperformance. We demonstrate that the finite sample performance of ITC-SIS is accurate and fast through various simulation designs.

**Conclusion:**

ITC-SIS serves as a good alternative method to detect disease susceptibility loci for clinic genomic data.

## Background

Ultrahigh dimensional data with binary response and categorical features has become increasingly prevalent in various fields. Applications using such data exist in genome-wide association studies (GWAS), medical imaging, finance, text mining, among others [1, 2]. The most prevailing gene selection approaches used in genome-wide association studies consider an association of each of the genetic variant using univariate models (i.e, single-SNP models); however, they evaluate the association of each SNP in isolation from the others and hence ignore combined joint effects of multi-loci [3–7]. As a matter of fact, most complex diseases are reported to be mediated through multiple genetic variants, each conferring a small or moderate effect with low penetrance, which obscures the individual significance of each variant [8, 9]. Furthermore, 70−−80*%* of genomes showing regions of high linkage disequilibrium (LD), which is the nonrandom association of alleles at nearby loci [10–12]. Malo et al. (2008) claimed that single-SNP approaches failed to differentiate truly influential SNPs from spurious SNPs that were merely in LD with the influential SNPs [13]. Therefore, although widely used in GWAS data analyses for its simplicity, single-SNP models have limited power and yield both high false-positive and false-negative results [13–15].

Many joint models can be applied to analyze the association between categorical features and binary response if the dimension is moderate. For example, random forests [16, 17], *k*-nearest neighbors [18], and support vector machines [19, 20], etc. However, these methods may lose power or become increasingly unstable, and hence intractable, as the dimension of feature space becomes ultrahigh [21]. To fully address the joint effect of multi-loci together with confounding caused by LD for high dimensional data, the penalized regression approaches have been well established and widely used in gene selections [13, 22, 23]. Through intensively investigating 48 different settings with varied LD strength, minor allele frequency, and dimensionality, Michelle et al. empirically verified that the running time and selection success rates decreased dramatically as the dimension of SNPs is greater than 1000 and even worse for 10,000 when applying a multiple logistic penalized ridge regression to gene selection [15]. Furthermore, the computational expediency, statistical accuracy and theoretical disadvantages of penalized regression approaches were concerned for ultrahigh dimensional data analyses [21, 24], which represent the true need for genome-wide association studies. As commented by Fan et al. [21, 25], the fundamental challenges of big data come from the accumulation of aggregate error rates due to a preponderance of noise features. Actually the majority of the information in ultrahigh dimensional data is represented by only a small amount of truly influential features.

Feature screening has garnered considerable attention in recent statistic literature. It filters out a substantial amount of noise features to truly reflect the sparsity principle of the ultrahigh dimensional data. In their seminal paper, Fan and Lv established the underpinnings for what they termed sure independent screening (SIS) and introduced the conceptual framework of the bulk of feature screening literature that come thereafter [26]. Even though many approaches stemming from [26] relaxed the model specification assumptions (see an overview in [27]), many existing SIS-based procedures still tacitly require that the feature variables and the response are continuous [28–31]. Notably, this implicit presupposition of continuity of the variables can be limiting in several application directions, for example in GWAS area.

Motivated by a polycystic ovary syndrome (PCOS) data with 4,099 observations (1,043 cases and 3,056 controls) and 731,442 single nucleotide polymorphisms (SNPs) (*p*>>*n*), in this article we propose a new feature screening method based on the iterative trend correlation (we call it ITC-SIS for short). We prove that trend correlation based strong independence screening (TC-SIS) satisfies the strong screening consistency property under a set of reasonable conditions, which is a much stricter criterion than the generally proved sure screening property. Since TC-SIS is a marginal approach, the iterative process on TC-SIS is applied to detect the multi-loci effect each having weak main effects, and separate the individual variants that are truly influential from those confounding spurious variants that are irrelevant to the response but highly correlated with the causative loci due to LD.

There are three existing methods in the statistic feature screening literature that also admit a binary response and categorical features for ultrahigh dimensionality: the maximum marginal likelihood estimator based approach (MMLE-SIS) [32], the distance correlation based approach (DC-SIS) [24], and the Pearson’s chi-squared test based approach (PC-SIS) [2]. We compare TC-SIS with these three most relevant methods and demonstrate that TC-SIS has agreeable accuracy and speed in handling the motivated setting with categorical feature and binary outcome through various finite sample simulation studies. We also demonstrate the ITC-SIS indeed improves TC-SIS through iterative process.

## Methods

### 2.1 Some preliminaries

Trend correlation was applied to measure association between two categorical variables [33, 34]. Specifically, let *Y* be the response variable and *X*_*j*_, *j*=1,…,*p*, be the *j*^*th*^ categorical feature variable. Define $v_{k}^{(j)}$ be the numeric score assigned to each level of *X*_*j*_, where *k*=1,…,*K*_*j*_. Here *p* is the total number of features and *K*_*j*_ is the number of levels for *X*_*j*_, for which we allow for various number of levels for different features. Let *m*=0,1 be the encoding of the case-control response *Y*.

The trend correlation *ϱ*_*j*_ between the response *Y* and feature *X*_*j*_ is defined as follows [35]

1 $$ \varrho_{j} = \frac{\left|\sum\limits_{k = 1}^{K_{j}}\sum\limits_{m = 0}^{1}(v_{k}^{(j)}-\mathbb{E} X_{j})(m-\mathbb{E}{Y}){p}_{km}^{(j)}\right|}{\sqrt{\left(\sum\limits_{k = 1}^{K_{j}}(v_{k}^{(j)}-\mathbb{E}{X_{j}})^{2}{p}_{k}^{(j)}\right)\left(\sum\limits_{m = 0}^{1}(m-\mathbb{E}{Y})^{2}{p}_{m}\right)}},   $$

where

$$\quad{p}_{k}^{(j)} = \mathbb{P}(X_{j} = k),\quad {p}_{m} = \mathbb{P}(Y = m), \quad {p}_{km}^{(j)} = \mathbb{P}(X_{j} = k, Y = m)$$

are the frequencies of each level of the feature, response, and individual cell of the contingency table, respectively. We are motivated to utilize *ϱ* as a feature screening procedure because it possesses the rather salient property of being equal to zero if and only if the two involved variables are independent [35].

### 2.2 A new independence ranking and screening procedure

Here we describe the details of the proposed TC-SIS screening procedure. For a sample of *n* observations, we will denote the sample mean score of *X*_*j*_ by ${\bar {v}^{(j)}}$ and sample mean of *Y* as $\bar {Y}$. We then estimate the trend correlation between *X*_*j*_ and *Y* as [35]

2 $$ \hat{\varrho}_{j}=\frac{\left|\sum\limits_{k = 1}^{K_{j}}\sum\limits_{m = 0}^{1}(v_{k}^{(j)}-\bar{v}^{(j)})(m-\bar{Y})\hat{p}_{km}^{(j)}\right|}{\sqrt{\left(\sum\limits_{k = 1}^{K_{j}}(v_{k}^{(j)}-\bar{v}^{(j)})^{2}\hat{p}_{k}^{(j)}\right)\left(\sum\limits_{m = 0}^{1}(m-\bar{Y})^{2}\hat{p}_{m}\right) }},   $$

where $\hat {p}_{km}^{(j)}$, $\hat {p}_{k}^{(j)}$, and $\hat {p}_{m}$ represent the sample proportions that are used to estimate the corresponding population proportions ${p}_{km}^{(j)}$, ${p}_{k}^{(j)}$, and *p*_*m*_, respectively.

When the features are ordinal, we can interpret $\hat {\varrho }_{j}$ as estimating the linear *trend* between *X*_*j*_ and *Y*, e.g., an increase in the observed level of *X*_*j*_ tends to be associated with decreasing or increasing levels of *Y* [35]. Therefore, it is suggested that the ordering of and the distance between the $v_{k}^{(j)}$ scores conform to those of the categorical levels.

The sparsity principle of ultrahigh dimensional data indicates that only a small number of the features truly influence the response. Define $\mathcal {S}_{F}$ as the set of the full model, i.e., all features in the candidate pool. Let $\mathcal {S}$ be a subset of $\mathcal {S}_{F}$, i.e., an arbitrary model under consideration.

We use $\hat {\varrho }_{j}$ as a marginal utility to rank the importance of each *X*_*j*_ according to its associations with the response, where higher $\hat {\varrho }_{j}$ values correspond to stronger association. Note that $\hat {\varrho }_{j}$ is non-negative because the absolute values are used in the numerator. As a output of the TC-SIS feature screening procedure, the selected model are given by

3 $$ \widehat{\mathcal{S}} = \{j: \hat{\varrho}_{j} > c, for ~~1\leq j \leq p\},   $$

where *c* is a pre-specified threshold value.

The aim of feature screening is to select the true model or at least select a model that contains the true model. As a matter of further notation, we will denote the true model by $\mathcal {S}_{T}$ and the selected model output from TC-SIS by $\widehat {\mathcal {S}}$.

### 2.3 Theoretical properties

In this section the theoretical properties of the proposed independence screening procedure TC-SIS will be studied. We first define two conditions to facilitate the technical proofs:

1. *Bounds on the standard deviations*. Assume that there exists a positive constant *σ*_min_ such that for all *j*,
  $$ { \min(\sigma_{j},\sigma_{Y})\geq\sigma_{min}>0}. $$
  This excludes unusual or unreasonable features that are constant and hence have a standard deviation of zero. It should further be noted that an upper bound on *σ*_*j*_ and *σ*_*Y*_ can also be obtained, by use of Popoviciu’s inequality on variances (see [36]):
  $$ {\max(\sigma_{j},\sigma_{Y})\leq \sigma_{\text{max}} = \text{max}\left\{\frac{1}{2},~ \sqrt{\frac{1}{4}\left(\max(v_{k}^{(j)})-\min({v}_{k}^{(j)})\right)}\right\}.} $$
2. *Lower bound on the covariance*. Assume that *ϱ*_*j*_=0 for any $j \not \in \mathcal {S}_{T}$. Assume that there exists a positive constant *ω*_min_ such that
  $$\min_{j \in \mathcal{S}_{T}} \left|\sum\limits_{k = 1}^{K_{j}}\sum\limits_{m = 0}^{1}(v_{k}^{(j)}-\mathbb{E} X_{j})(m-\mathbb{E}{Y}){p}_{km}^{(j)}\right| \geq \omega_{\min}>0,$$
  which indicates that the correlation between each truly influential feature and the response is not trivial.

When these two conditions are satisfied, we can establish the following theorems that support the *strong screening property* for the TC-SIS procedure.

#### **Theorem 1**

(*Sure Screening Property*). Under condition (C1) and removing from (C2) only the assumption that *ϱ*_*j*_=0 for any $j \not \in \mathcal {S}_{T}$, there exists a positive constant *c*>0 such that

$$\mathbb{P}{\left(\mathcal{S}_{T} \subseteq \widehat{\mathcal{S}}\right)} \rightarrow 1 \text{ as} n \rightarrow \infty.$$

(However, $\mathbb {P}\left (\widehat {\mathcal {S}} \subseteq \mathcal {S}_{T}\right)$ may not converge to 1 as *n* approaches infinity).

#### **Theorem 2**

(*Strong Screening Consistency*). Given conditions (C1) and (C2), there exists a positive constant *c*>0 such that

$$\mathbb{P}\left(\widehat{\mathcal{S}} = \mathcal{S}_{T}\right) \rightarrow 1 \text{ as} n \rightarrow \infty.$$

The property of strong screening consistency is much harder to achieve than the (weak) sure screening property because it not only guarantees that the true model is contained in the selected subset, but also ensures that the selected subset is the minimum one containing the true model asymptotically. The proofs of these two theorems are presented in the Supplement file. In addition to the aforementioned two theorems, we also draw two corollaries, which are not themselves related to sure screening, but they are nevertheless important conclusions related to the screening criterion of the TC-SIS method.

#### **Corollary 1**

There exists a positive value *ϱ*_min_>0 such that for any $j \in \mathcal {S}_{T}$, we have *ϱ*_*j*_>*ϱ*_min_>0. This will be shown in Step 1 of the proofs of Theorems 1 and 2.

#### **Corollary 2**

The estimator ${\hat {\varrho }_j}$ converges *uniformly* in probability to *ϱ*_*j*_. In other words,

$$\mathbb{P}\left(\max_{1 \leq j \leq p}|{\hat{\varrho}_j} - \varrho_{j}| > \varepsilon\right) \rightarrow 0 \quad \text{ as} n\rightarrow\infty$$

for any *ε*>0. This will be shown in Step 2 of the proofs of Theorems 1 and 2.

### 2.4 Iterative process

Although the proposed TC-SIS approach is powerful at filtering out noise and selecting the truly influential features for high dimensional setting of *p*>*n*, it may neglect some important features that are jointly associate with the response but have weak individual effects. Furthermore, as a marginal approach, it may rank highly some unimportant SNPs that are spuriously correlated with the response due to their strong collinearity with other influential features [26, 37]. To overcome these shortcomings, we use the iterative process to address possible complex situations of SNPs that can exist.

The main difference between TC-SIS and ITC-SIS is that TC-SIS finalizes the first *d* members of $\mathbf {X}_{\hat {\mathcal {S}}}$ by only one step while IDC builds up $\mathbf {X}_{\hat {\mathcal {S}}}$ gradually with several steps [37], i.e. $\mathbf {X}_{\hat {\mathcal {S}}}=\mathbf {X}_{\hat {\mathcal {S}}_{1}} \bigcup \ldots \bigcup \mathbf {X}_{\hat {\mathcal {S}}_{k}}$, with *d*=*d*_1_+*d*_2_+…+*d*_*k*_, where $\mathbf {X}_{\hat {\mathcal {S}}_{i}}$ stands for the SNPs selected at *i*^*th*^ step and *d*_*i*_ is the number of SNPs for each set $\mathbf {X}_{\hat {\mathcal {S}}_{i}}$, for *i*=1,…,*k*. The main idea of ITC-SIS is to iteratively adjust residuals obtained from regressing all remaining SNPs onto the selected ones contained in $\mathbf {X}_{\hat {\mathcal {S}}}$. Regressing unselected on selected, and adjusting residuals, effectively breaks down original complex correlation structure among SNPs. The iterative steps of ITC-SIS can be summarized as:

- Step 1: Use $\hat {\varrho }_{j},~j=1,\ldots,p$ to rank all SNPs based on their individual trend correlations with the response, and then input the first *d*_1_ members into $\mathbf {X}_{\hat {\mathcal {S}}}$$\left (\text {i.e.} \mathbf {X}_{\hat {\mathcal {S}}}=\mathbf {X}_{\hat {\mathcal {S}}_{1}}\right)$, where *d*_1_<*d*.
- Step 2: Define $\mathbf {X}_{r}=\left \{I_{n}-\mathbf {X}_{\hat {\mathcal {S}}}\left (\mathbf {X}_{\hat {\mathcal {S}}}^{T}\mathbf {X}_{\hat {\mathcal {S}}}\right)^{-1}\mathbf {X}_{\hat {\mathcal {S}}}^{T}\right \}\mathbf {X}_{\hat {\mathcal {S}}}^{C}$, where $\mathbf {X}_{\hat {\mathcal {S}}}^{C}$ is the complement set of $\mathbf {X}_{\hat {\mathcal {S}}}$. Then choose the second *d*_2_ members into $\mathbf {X}_{\hat {\mathcal {S}}}$$\left (\text {i.e.} \mathbf {X}_{\hat {\mathcal {S}}}=\mathbf {X}_{\hat {\mathcal {S}}_{1}} \bigcup \mathbf {X}_{\hat {\mathcal {S}}_{2}}\right)$ using TC-SIS to rank all candidates of **X**_*r*_ for *Y*, where *d*_1_+*d*_2_≤*d*.
- Step 3: repeat step 2 until the size of $\mathbf {X}_{\hat {\mathcal {S}}}$ reaches the pre-specified number *d*.

See more details of the iterative process from Zhong et al. [37].

We refer the reader to an example of a single gene with strong SNP.

## Results

In this section, we assess the performance of TC-SIS by four empirical Monte Carlo simulation studies under various designs, and also a real data analysis examining the genome-wide association studies on PCOS affection status. We evaluate the performance of the screening procedures through the following three criteria [24]:

- Average Minimum Model Sizes $\left (|\widehat {\mathcal {M}}|\right)$: The average of the minimum number of features that are required by each screening procedure to select all truly influential features across all simulation replicates. The closer to the true model size for the estimated $|\widehat {\mathcal {M}}|$ is, the better the screening procedure is determined to be.
- Individual Success Rates $\left (P_{X_{i}}\right)$: The proportion of each truly influential feature is correctly selected by the screening method within the threshold *d* across all simulation replicates. This requires that the screening score of each truly influential feature ranks within the top *d* among all *p* features.
- Simultaneous Success Rates (*P*_*a*_): The proportion of replicates in which all of the truly influential features are simultaneously selected by the screening method within the threshold *d*. This requires that the screening scores of all truly influential features rank within the top *d* among all *p* features. The closer to one that this proportion is, the better the screening procedure is determined to be.

### 3.1 Simulation study 1

In this Simulation Study we directly adopt a published real genome-wide association data collected from rice accessions, including 36,901 SNPs and 272 samples [38]. We set the first five SNPs as the truly influential ones, and notice that the correlations among them are complex, ranging from 0.19 (slightly correlated) to 1 (perfectly correlated). The remaining 36,896 SNPs serve as confounding noise, representing a complicated genome simulation setting. We design a joint effect of these five loci by generating the response from a multiple logistic regression model as

$$log \frac{P(Y=1)}{1 - P(Y=1)} = X\beta + \epsilon,$$

where the residual term *ε* is generated from *N*(0,1). The coefficients of the five influential SNPs, *β*_*i*_(*i*=1,...5), are randomly selected from a mixed Gaussian distribution:

$$\beta_{i} \sim N(5 Z_{i}, 1), \quad\quad \text{where} \quad Z_{i} =\{-1,1\}\sim \text{Bernoulli}(0.5). $$

In Simulation Study 1, we replicate each simulation 100 times, and compare four methods: DC-SIS, MMLE-SIS, TC-SIS, and ITC-SIS. For ITC-SIS, *d*_1_ is set to be 6, *k*=2, and *d*_2_=*d*−*d*_1_. The results are summarized in Table 1. Given each of the same thresholds, TC-SIS achieves higher individual success rates than DC-SIS and MMLE-SIS. However, the simultaneous success rates of all the first three methods are all very low (close to zero) because they are trapped by a couple of influential loci each having very weak individual effect but associating with the response by joint effects with other loci. Therefore, a large amount of confounding SNPs that are not actually associated with the response but appear to be important because of their high LDs with the other loci act as a role to confuse the individual/marginal approaches (the first three) to include 13000 SNPs on average to locate all of the five true loci. Compared to these three individual approaches, ITC-SIS requires an average model size of only 31.69 to simultaneously select all of the five truly influential loci from 36,901 SNP candidates across the 100 simulation replicates. It is a striking improvement because 31.69 is only 2 thousandth (0.002) of the model size needed by the first three approaches. It indicate that the iterative process is very effective in successfully detecting the true multi-loci without being trapped by spurious associations caused by LD. The running time of one replicate for ITC-SIS is around 11 seconds on a MacBook Pro with 2.2 GHz Intel Core i7 and 16GB RAM.

**Table 1:** Success rates of four feature screening approaches in selecting each and all truly influential feature *X*_*j*_ within thresholds *d*=20,40,60 for Simulation Study 1

*d*=20								
	*P*_*a*_	$|\widehat {\mathcal {M}}|$	$P_{X_{1}}$	$P_{X_{2}}$	$P_{X_{3}}$	$P_{X_{4}}$	$P_{X_{5}}$	Run Time
DC-SIS	0	20927.89	0.45	0.46	0.27	0.60	0.48	
MMLE-SIS	0	13982.14	0.77	0.78	0.25	0.39	0.78	
TC-SIS	0.03	28202.48	0.99	0.67	0.29	0.77	0.45	5.042s
ITC-SIS	0.22	31.69	1.00	0.99	0.47	0.76	0.99	11.025s
*d*=40								
	*P*_*a*_	$|\widehat {\mathcal {M}}|$	$P_{X_{1}}$	$P_{X_{2}}$	$P_{X_{3}}$	$P_{X_{4}}$	$P_{X_{5}}$	Run Time
DC-SIS	0	20927.89	0.54	0.55	0.30	0.61	0.56	
MMLE-SIS	0	13982.14	0.79	0.79	0.27	0.39	0.79	
TC-SIS	0.03	28202.48	0.99	0.67	0.31	0.77	0.45	5.042s
ITC-SIS	0.89	31.69	1.00	1.00	0.95	0.94	1.00	11.025s
*d*=60								
	*P*_*a*_	$|\widehat {\mathcal {M}}|$	$P_{X_{1}}$	$P_{X_{2}}$	$P_{X_{3}}$	$P_{X_{4}}$	$P_{X_{5}}$	Run Time
DC-SIS	0	20927.89	0.61	0.62	0.31	0.62	0.63	
MMLE-SIS	0	13982.14	0.79	0.79	0.27	0.39	0.79	
TC-SIS	0.03	28202.48	0.99	0.67	0.31	0.78	0.45	5.042s
ITC-SIS	0.94	31.69	1.00	1.00	0.97	0.97	1.00	11.025s

For each of the following three simulation designs, we fix the sample size, *n*, to be 200 and set the number of features, *p*, to be 5,000. We replicate each simulation 500 times and compared four approaches, MMLE-SIS, DC-SIS, PC-SIS, and TC-SIS. To be fair, we compared only these four marginal approaches without applying iterative process for any of them.

### 3.2 Simulation study 2

Each observation of the response, *Y*_*i*_, will be generated by a Bernoulli process with $\mathbb {P}(Y = 1) = p_{y},$ where *p*_*y*_∼Unif(0.05,0.95) is chosen anew for each replicate of the simulation. We design the first ten features to be truly associated with the response *Y*, i.e., *S*_*T*_={1,…,10}. Similar to Example 1 of [2], we generate these first ten features as

$$\left\{ X_{ij} \mid Y_{i} = m \right\} \sim \text{Binomial}\left(2, \pi_{mj}\right);~m=0,1;~j\in S_{T},~i=1,\ldots,n,$$

with the values of *π*_*mj*_ being given by Table 2. This means that each causative *X*_*j*_ will take on values of 0, 1, or 2 (representative of three ordinal levels, with 0≺1≺2). For any *j*∉*S*_*T*_, we generate *X*_*j*_∼Binomial(2,*π*_*j*_) with *π*_*j*_∼Unif(0.05,0.95). The value of *π*_*j*_ is chosen anew with each replicate of the simulation. This means that these non-causative features will have no association with *Y*.

**Table 2:** Values of *π*_*mj*_ used to simulate data in Simulation Study 2

	*π*_*m*1_	*π*_*m*2_	*π*_*m*3_	*π*_*m*4_	*π*_*m*5_	*π*_*m*6_	*π*_*m*7_	*π*_*m*8_	*π*_*m*9_	*π*_*m*,10_
*Y*=0	0.3	0.4	0.6	0.7	0.2	0.4	0.3	0.8	0.4	0.2
*Y*=1	0.6	0.1	0.1	0.4	0.8	0.7	0.9	0.2	0.7	0.6

The results are summarized in Table 3. For this simulation, TC-SIS results in the smallest average model size of 54.674 to contain all the ten truly influential features, which is ten features less than the next closest method (DC-SIS for 64.990). The average minimum model sizes of PC-SIS and MMLE-SIS nearly double or triple that required by TC-SIS, respectively. In the case of TC-SIS versus MMLE-SIS, the individual success rates are at times nearly fourfold more favorable towards our method.

**Table 3:** Success rates of four feature screening approaches in selecting each truly influential feature *X*_*j*_ within thresholds *d*=15, respectively for Simulation Study 2

*d*=15											
	$|\widehat {\mathcal {M}}|$	*X*_1_	*X*_2_	*X*_3_	*X*_4_	*X*_5_	*X*_6_	*X*_7_	*X*_8_	*X*_9_	*X*_10_
MMLE-SIS	150.340	0.384	0.746	0.756	0.404	0.822	0.844	0.354	0.742	0.320	0.400
DC-SIS	64.990	0.900	0.984	0.990	0.898	0.998	0.998	0.894	0.984	0.888	0.894
PC-SIS	93.018	0.862	0.974	0.980	0.864	0.994	0.998	0.860	0.966	0.854	0.862
TC-SIS	54.674	0.916	0.988	0.994	0.922	1.000	0.998	0.912	0.982	0.904	0.908

### 3.3 Simulation study 3

Inspired by the concept of discretization of a continuous random variable as found in Example 3 of [2], we connect the influential features with the response via an indirect way. Similar to Simulation Study 2, we generate the response *Y*_*i*_ from a Bernoulli process with $\mathbb {P}(Y_{i} = 1) = p_{y},$ where *p*_*y*_∼unif(0.05,0.95) is again chosen anew for each replicate of the simulation. Given *Y*_*i*_=*m*, we generate a latent variable *Z*_*ij*_ independently distributed as *N*(*Y*_*i*_,1) for the first ten truly influential features *j*∈*S*_*T*_. The first ten influential features *X*_*ij*_ are then discretized from *Z*_*ij*_ based on the cutoffs (*κ*_*Lj*_,*κ*_*Uj*_) listed in Table 4 as:

$$X_{ij} = \left\{\begin{array}{lll} 0 & \text{if} Z_{ij} < \kappa_{Lj},\\ 1 & \text{if} \kappa_{Lj} \leq Z_{ij} \leq \kappa_{Uj}, ~i=1,\ldots,n;~~j=1,\ldots,10.\\ 2 & \text{if} Z_{ij}>\kappa_{Uj}. \end{array}\right.$$

**Table 4:** Values of (*κ*_*Lj*_,*κ*_*Uj*_) used to simulate data in Simulation Study 3

	*X*_1_	*X*_2_	*X*_3_	*X*_4_	*X*_5_	*X*_6_	*X*_7_	*X*_8_	*X*_9_	*X*_10_
*κ*_*Lj*_	0	0	0.2	0	-0.2	0.2	0	0.1	-0.2	0.2
*κ*_*Uj*_	0.7	1	0.8	0.9	1.2	1	1	1	1.2	0.8

These cutoffs in Table 4 are set to establish weaker associations between the response *Y* and each of the influential feature to increase the difficulty level in recognizing the true model. It should be noted that this method of generating the truly influential features results in a trend association between each of the truly influential feature and response: namely, lower values of *X*_*j*_ are associated with *Y*=0 and higher values of *X*_*j*_ are associated with *Y*=1. For any *j*∉*S*_*T*_, we generate *X*_*j*_∼Binomial(2,*π*_*j*_) with *π*_*j*_∼Unif(0.05,0.95).

The results are summarized in Table 5. TC-SIS here results in the smallest average minimum model size of 112.627 to get the true model. This leads us to the conclusion that TC-SIS does a better job than other approaches at avoiding ballooning models. Of especial note here, MMLE-SIS fails on average to produce a selected model smaller than the sample size of *n*=200 and its success rates are at many times less than 0.1 (versus 0.8 of TC-SIS). These results demonstrate the capability of TC-SIS to obtain excellent results when trend correlation exists between the feature and response. The performance of DC-SIS is comparable in the success rates but at the cost of a relatively larger model.

**Table 5:** Success rates of four feature screening approaches in selecting each truly influential feature *X*_*j*_ within thresholds *d*=15, respectively for Simulation Study 3

*d*=15											
	$|\widehat {\mathcal {M}}|$	*X*_1_	*X*_2_	*X*_3_	*X*_4_	*X*_5_	*X*_6_	*X*_7_	*X*_8_	*X*_9_	*X*_10_
MMLE-SIS	508.672	0.072	0.060	0.066	0.078	0.554	0.054	0.388	0.098	0.032	0.204
DC-SIS	125.258	0.876	0.884	0.886	0.904	0.886	0.880	0.880	0.910	0.878	0.906
PC-SIS	171.829	0.820	0.806	0.816	0.842	0.876	0.806	0.876	0.830	0.820	0.858
TC-SIS	112.627	0.876	0.876	0.882	0.904	0.924	0.874	0.920	0.906	0.878	0.900

### 3.4 Simulation study 4

In this simulation, we generate the sample data using a logistic regression model, which is the fundamental basis of MMLE-SIS. We first generate each feature *X*_*j*_ (1≤*j*≤*p*) by uniformly sampling from the set {0,1,2} with equal probability. We then connect a binary response *Y* with the first five features by letting

$$L_{i} = \sum_{j = 1}^{5} \left[I \left(X_{ij} = 0 \right) \times \beta_{X_{j}=0} + I \left(X_{ij} = 1 \right) \times \beta_{X_{j}=1} + I \left(X_{ij} = 2 \right) \times \beta_{X_{j}=2}\right],$$

and using

$P \left (Y_{i} = 1|X_{i} \right) = \frac {1}{1 + \text {exp}\left (-L_{i}\right)},$

to sample the binary response. The coefficients $\beta _{X_{j} = k}$ are as given in Table 6. The results are summarized in Table 7. For this example, we once again obtain a smaller required average minimum model size than DC-SIS and PC-SIS (46.470 for DC-SIS, 93.270 for PC-SIS, as compared to 41.976 for TC-SIS). Unlike the first two examples, MMLE-SIS recoups its earlier collapses and matches TC-SIS nearly perfectly in both success rates and average minimum model sizes in Example 3. Although it is the specific strength of MMLE-SIS to handle logistic regression model, TC-SIS still produces results abreast with that of MMLE-SIS. In addition, it should be noted that since MMLE-SIS requires solving an optimization problem to produce its screening statistics, TC-SIS is significantly faster in computational run time. Thus, when run time is an issue, we suggest the use of TC-SIS over MMLE-SIS, even when the logistic regression model holds.

**Table 6:** Values of $\beta _{X_{j} = k}$ used to simulate data in Simulation Study 4

	$\beta _{X_{1}}$	$\beta _{X_{2}}$	$\beta _{X_{3}}$	$\beta _{X_{4}}$	$\beta _{X_{5}}$
*X*_*j*_=0	0	-5	2	-6	1
*X*_*j*_=1	3	-3	4	-4	3
*X*_*j*_=2	5	-1	6	-2	5

**Table 7:** Success rates of four feature screening approaches in selecting each truly influential feature *X*_*j*_ within thresholds *d*=15, respectively for Simulation Study 4

*d*=15						
	$|\widehat {\mathcal {M}}|$	*X*_1_	*X*_2_	*X*_3_	*X*_4_	*X*_5_
MMLE-SIS	41.934	1.000	0.856	0.868	0.842	0.870
DC-SIS	46.470	1.000	0.860	0.850	0.838	0.866
PC-SIS	93.270	1.000	0.794	0.758	0.778	0.790
TC-SIS	41.976	1.000	0.860	0.858	0.842	0.862

### 3.5 Real data analyses

We apply the proposed ITC-SIS screening procedure to a clinical dataset pertaining to the genome-wide association studies on the PCOS affection status (dbGaP Study Accession: https://www.ncbi.nlm.nih.gov/projects/gap/cgi-bin/study.cgi?study_id=phs000368.v1.p1). This data consists of 4,099 subjects (1,043 cases and 3,056 controls) and 731,442 SNPs. The goal of this analysis is to identify the most influential susceptibility loci that affect PCOS status for European Caucasian population. The response for this data is PCOS affection status (binary) and the features are the encoded SNP genotype values (categorical), which exactly represents a problem that the ITC-SIS is originally motivated.

**Fig. 1:**
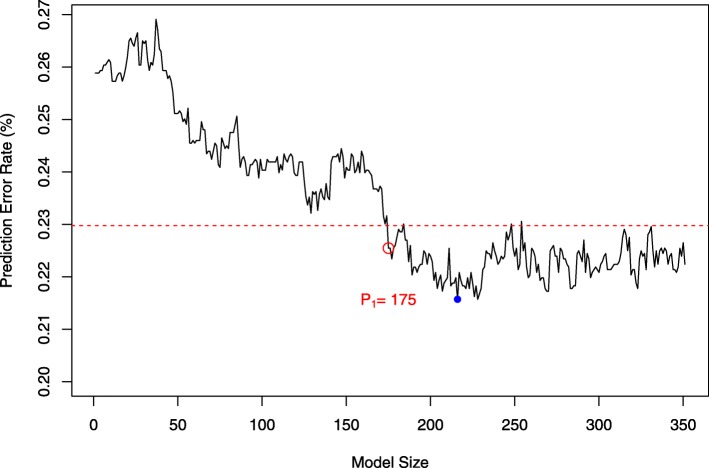
The MSPE used to select *d*_1_ in Real Data Analyses

We removed rare alleles that have minor allele frequency (MAF) less than 0.1 before performing the ITC-SIS. To determine the optimal value *d*_1_ for the fist iterative process, we investigate each value among a set of *d*_1_=1,2,…,3[*n*^4/5^/*log*(*n*^4/5^)]=351 and check the mean square prediction error (MSPE). The prediction is assessed by a cross validation process, with 75% of the observed data for training and 25% for testing. As shown in Fig. 1, as the model size increases, the MSPE first drops and then stays flat. We choose *d*_1_ as the minimum model size whose MSPE falls into one standard deviation plus the minimum MSPE. As demonstrated in Fig. 1, we select *d*_1_=175 SNPs in the first iteration, *d*_2_=351−175=176 SNPs in the second iteration, and number of iterations *k*=2. The remaining 731,091 SNPs are filtered out as noise.

After the completion of ITC-SIS, high dimensionality is not an issue any more (*n*=4099,*d*=351). Then we apply the multiple logistic regression model to estimate the multi-loci joint effects of these promising candidates, which is able to assess the significance level of each SNP through *p*-values. We compare the results of multiple logistic regression after integrating it with each of the DC-SIS, TC-SIS, and ITC-SIS screening process (see Table 8). Three model selection criteria are used: model size, Akaike’s Information Criterion (AIC), and misclassification rate, which is computed as the percentage of incorrectly predicted affection status after applying the multiple logistic regression model to fit the selected 351 SNPs that are selected by each screening procedure.

**Table 8:** Model Selection of Three approaches Applied in Real Data Analyses

Two-stage Method	Model size	AIC	Misclassification Rate
DC-SIS + Multiple Logistic Regression	70	4188.33	21.74%
TC-SIS + Multiple Logistic Regression	86	3601.02	19.51%
ITC-SIS (*d*_1_=175) + Multiple Logistic Regression	88	3581.96	19.27%

**Fig. 2:**
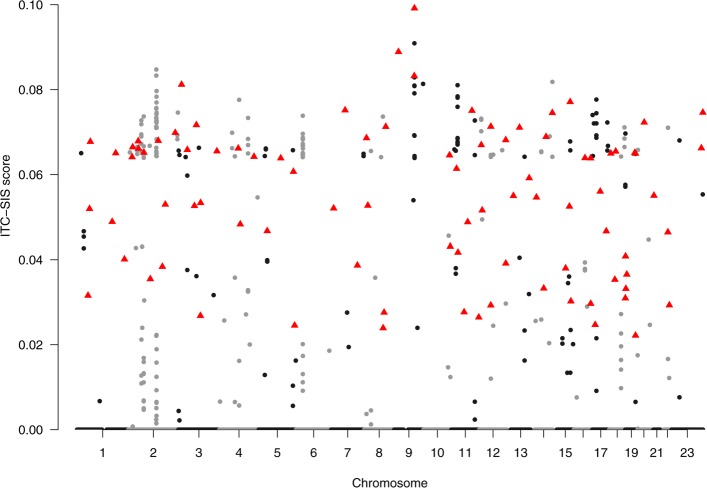
The Manhattan plot of the ITC scores in Real Data Analyses

As expected, ITC-SIS + multiple logistic regression yields the best model with the smallest misclassification rate and the smallest AIC. It suggests 88 influential SNPs that was highlighted as red triangle in Fig. 2. Figure 2 is very different from the traditional Manhattan plot that was obtained by single-SNP approaches from several aspects: 1) It is much less dense because the iterative feature screening process dramatically shrinks noise SNPs into zero that makes 99.9% of SNPs disappeared from current plot. The noise SNPs built up a very tall and dense base in traditional Manhattan plot. 2) Unlike traditional Manhattan plot, the vertical axis demonstrates ITC-SIS scores instead of *p*-values. As a two-stage approach that explore the complex structure of the data, individual *p*-value will not be meaningful in this plot. 3) It separates important SNPs from noise SNPs in a much striking way. Specifically, noise SNPs are in the bottom line that makes the selected SNPs substantially stand out. 4) It revolutionizes the traditional selection rule that only select the significance from the top of the traditional Manhattan plot. You may wonder why several SNPs with very small scores are selected but others with higher scores are not selected. It actually reflects the joint effects of multi-loci and confounding issue of LD described in early sections of this article. Specifically, PCOS is a complex disease that are affected by multi-loci each having small individual effects, meanwhile many noise SNPs may have strong signals because they are in strong multicollinearity with other truly influential SNPs but they actually do not directly associated with the disease. To show the data-driven nature of our proposed approach, we refer the readers to a publication that also used iterative feature screening for GWAS data but worked on a single-gene trait with strong individual effects. As you can see, their SNPs were selected based on scores locating on the top of the Manhattan plot [15].

There are over 50 genes being located from these 88 informative SNPs. Additional file 1: Table 1 in the Supplement file summarizes the estimated ITC score, $\hat {\beta }$ coefficient, *p*-value, corresponding gene name, Allele type, and detailed position for each of the 53 selected influential SNPs that could locate nearby genes. In addition to confirming many genes (*FSHR, LHCGR, C9orf3, RPS26, RAB5B, SUOX, ERBB3, TOX3, ApoB, ROBO2, NEIL2*) that were reported to be directly associated with PCOS, we also detect several new genes. Specifically, we find that the SNP rs7559066 located at Chr 2 lies within the *FSHR* gene and the SNP rs12235316 located at Chr 9 lies within the *C9orf3* gene. The *FSHR* and *C9orf3* gene have been reported by numerous studies to be strongly associated with PCOS in women and erectile dysfunction in men for both Han Chinese population [39] and European Caucasian population [40], which appear in individuals who have either inadequate or excessive amounts of sexual hormones. Adolescent girls with obesity and PCOS were found to have elevated fasting and postprandial plasma TG and *ApoB*-lipoprotein remnants [41]. *ROBO2* gene were found differentially expressed between obese women without PCOS and obese women with PCOS [42]. *NEIL2* gene may help identify pathways that link specific PCOS related traits with greater metabolic risk [43]. *ERBB3* is T2D candidate gene, implicated in the process of female gamete generation and determining function of antigen-presenting cells [39].

The new genes detected from this data analyses could be found in Table 1 of the supplement file. To name a few, *TACR1* and *GASK1A* have broad expression in endometrium. *LTBP2* has broad expression in ovary (RPKM 21.5). *NR2C2* encodes a protein that belongs to the nuclear hormone receptor family [43]. *MSH6* and *BRCC3* were found to be relevant for PCOS related phenotypes by a new protein-protein interaction network analysis [44]. *NR2C2* encodes a protein that belongs to the nuclear hormone receptor family functioning in development, cellular differentiation and homeostasis. In summary this analysis confirm many genes that were reported to be associated with PCOS and also locate several new genes that are related to endometrium, hormones, organ growth, and cell division. Their functions in PCOS need further investigations by molecular and functional genetics.

## Discussion

In this paper, we propose a new feature screening procedure using trend correlation, whose finite performance is demonstrated via performing multiple simulation studies with various designs and also comparing with three other relevant extant approaches. We furthermore illustrate the performance of TC-SIS through real data analyses pertaining to genome-wide association studies on PCOS disease. We establish the strong screening consistency for this procedure when the number of features diverges exponentially with respect to the sample size. Strong screening consistency is much harder to achieve than the sure screening property, as it guarantees that not only the selected model *contains* the true model, but also that the selected model *equals to* the true model asymptotically.

The proposed TC-SIS method can be easily extended to a categorical response having greater than two levels if needed; however, we only consider binary *Y* here because this allows for some simplification of our notation and proofs. It has been noted that the choice of a threshold *d*, the number of SNPs to keep, is of importance in feature screening literature. Several methods have been proposed to determine such a threshold, e.g. [2, 29, 45, 46]. We follow the rule of thumb proposed by Liu et al. [47], and set the cutoff of model size as multiplier of *d*=[*n*^4/5^/*log*(*n*^4/5^)]. In the simulation study 1 (a harder case), we test the cutoffs *d*=20, 2*d*=40, and 3*d*=60 for *n*=272. We set *d*=15 when *n*=200 for all other simulation studies. In the real data example, we choose a model size 3*d*=351 when *n*=4099 to avoid missing influential candidate from the beginning, and then test the significance of these candidate SNPs by a joint model that performs well if high dimensionality is not an issue.

In addition to the general association detected by other methods, TC-SIS excels in exploring the *trend association* between the response and the features, e.g., larger feature values tends to be associated with larger (or conversely, smaller) response values in certain practices. Another appealing advantage that we observe from the simulation studies is the relative stability of TC-SIS (compare to other methods) in the face of a potentially large unbalance in the number of positive (*Y*=1) responses. TC-SIS assumes milder conditions than other approaches in that it neither requires any regression model structure nor assumes any specific distribution of the data.

## Conclusion

Detecting important multi-loci that are associated with the complex disease is challenging because each locus may have weak effect. The ITC-SIS following by a multiple regression model serves as a good alternative method to detect disease susceptibility loci for clinic genomic data. It confirms around ten genes that were reported to be associated with PCOS and also detects many new genes after scanning a high dimensional set of SNPs.

## Supplementary information

**Additional file 1** In the Supplementary Materials we present in full the proofs for Theorems 1 and 2 given at “Theoretical properties” section of the main text, and additional results from real data analyses.

## Data Availability

The program code for the current study are available from the corresponding author on reasonable request. The dataset is free download.

## Abbreviations

TC-SIS: Trend correlation based sure independence screening
ITC-SIS: Iterative based TC-SIS procedure
GWAS: Genome-wide association studies
PCOS: Polycystic ovary syndrome
LD: Linkage disequilibrium
