# Supplementary material for: Detecting PCOS susceptibility loci from genome-wide association studies via iterative trend correlation based feature screening
Source: BMC Bioinformatics. 2020 May 4;21:177. doi: 10.1186/s12859-020-3492-z (PMC7199379; doi:10.1186/s12859-020-3492-z)
Supplement: Supplementary file 1 — Additional file 1 In the Supplementary Materials we present in full the proofs for Theorems 1 and 2 given at “Theoretical properties” section of the main text, and additional results from real data analyses. [file 12859_2020_3492_MOESM1_ESM.pdf]

METHODOLOGY

“Detecting PCOS Susceptibility Loci from Genome-wide Association Studies Via Iterative Trend Correlation Based Feature Screening” - Supplementary Materials

Xiaotian Dai[1†], Guifang Fu[1*†] and Randall Reese[2†]

[*]Correspondence:
gfu@binghamton.edu
[1]Department of Mathematical Sciences, SUNY Binghamton University, Vestal, New York
Full list of author information is available at the end of the article
[†]All authors equally contributed to this article, and the authors are listed in the alphabetical order.

In the Supplementary Materials we present in full the proofs for Theorems 1 and 2 given at Section 4.3 of the main text. Before proceeding into the proofs, we will establish a few lemmas that will be used repeatedly in the proofs.

Appendix A: Some Lemmas

In reference to the numerator of the definition of $\hat{\varrho}_j$ given in Equation (3) of the main text, we define

$$\hat{\tau}_j = \left| \sum_{k=1}^{K_j} \sum_{m=0}^{1} (v_k^{(j)} - \bar{v}^{(j)})(m - \bar{Y})\hat{p}_{km}^{(j)} \right|$$

as an estimator of the covariance between X_j and Y (i.e., $\mathrm{Cov}(X_j, Y)$). Furthermore, in accordance with the denominator of the definition of $\hat{\varrho}_j$ given in Equation (3) of the main text, we define

$$\hat{\sigma}_j = \sqrt{\sum_{k=1}^{K_j} (v_k^{(j)} - \bar{v}^{(j)})^2 \hat{p}_k^{(j)}}$$

as an estimator of the standard deviation of X_j (i.e., σ_j). Similarly, we define

$$\hat{\sigma}_Y = \sqrt{\sum_{m=0}^{1} (m - \bar{Y})^2 \hat{p}_m}$$

as an estimator of the standard deviation of Y (i.e., σ_Y).

Lemma 1. $\hat{\tau}_j$ is a consistent estimator of $|\mathrm{Cov}(X_j, Y)|$.

Proof We begin by first showing that $\hat{\tau}_j$ is equal to the following estimator for $|\mathrm{Cov}(X_j, Y)|$:

$$\left| \frac{1}{n} \sum_{i=1}^{n} (X_{ij} - \bar{X}_j)(Y_i - \bar{Y}) \right|, \qquad (1)$$

where $\bar{X}_j = \frac{1}{n}\sum X_{ij}$ and $\bar{Y}$ is defined the same as before. Specific to the focus of this article, we know that $Y_i \in \{0,1\}$ and $X_{ij} \in \{v_1^{(j)}, v_2^{(j)}, \ldots, v_{K_j}^{(j)}\}$, and that $\bar{X}_j = \bar{v}^{(j)}$. Let n_{km} denote the number of observations satisfying $\{X_{ij} = v_k^{(j)}\}$ and $\{Y_i = m\}$, meaning we can write $\hat{p}_{km}^{(j)} = n_{km}/n$. We can now rewrite Statement (1) as follows:

$$\left|\frac{1}{n}\sum_{i=1}^{n}(X_{ij} - \bar{X}_j)(Y_i - \bar{Y})\right|$$

$$= \left|\frac{1}{n}\sum(X_{ij} - \bar{X}_j)(1 - \bar{Y}) - \frac{1}{n}\sum(X_{ij} - \bar{X}_j)(\bar{Y})\right|$$

$$= \left|\frac{1}{n}\left((v_1^{(j)} - \bar{v}^{(j)})(1 - \bar{Y})n_{11} + \cdots + (v_{K_j}^{(j)} - \bar{v}^{(j)})(1 - \bar{Y})n_{K_j1}\right) - \frac{1}{n}\left((v_1^{(j)} - \bar{v}^{(j)})(\bar{Y})n_{10} + \cdots + (v_{K_j}^{(j)} - \bar{v}^{(j)})(\bar{Y})n_{K_j0}\right)\right|$$

$$= \left|\frac{1}{n}\sum_{k=1}^{K_j}\sum_{m=0}^{1}(v_k^{(j)} - \bar{v}^{(j)})(m - \bar{Y})n_{km}\right|$$

$$= \left|\sum_{k=1}^{K_j}\sum_{m=0}^{1}(v_k^{(j)} - \bar{v}^{(j)})(m - \bar{Y})\hat{p}_{km}^{(j)}\right|$$

$$= \hat{\tau}_j.$$

As convenient, we will use the form (1) when discussing $\hat{\tau}_j$.

Secondly, we now show that $\hat{\tau}_j$ is a consistent estimator of $|\mathrm{Cov}(X_j, Y)|$. Expanding Statement (1) yields

$$\hat{\tau}_j = \left|\frac{1}{n}\sum X_{ij}Y_i - \frac{1}{n}\sum \bar{X}_j Y_i - \frac{1}{n}\sum X_{ij}\bar{Y} + \frac{1}{n}\sum \bar{X}_j\bar{Y}\right|. \tag{2}$$

By applying the weak law of large numbers we have

$$\frac{1}{n}\sum X_{ij}Y_i \xrightarrow{P} \mathbb{E}(X_jY),$$

$$\frac{1}{n}\sum \bar{X}_jY_i \xrightarrow{P} \mathbb{E}(X_j)\mathbb{E}(Y),$$

$$\frac{1}{n}\sum X_{ij}\bar{Y} \xrightarrow{P} \mathbb{E}(X_j)\mathbb{E}(Y),$$

$$\frac{1}{n}\sum \bar{X}_j\bar{Y} \xrightarrow{P} \mathbb{E}(X_j)\mathbb{E}(Y),$$

with each term converges in probability to its corresponding expectation. Hence we have by the Mann-Wald Theorem [1–3] (also known as the Continuous Mapping Theorem),

$$\begin{aligned}\hat{\tau}_j &\xrightarrow{P} |\mathbb{E}(X_jY) - 2\mathbb{E}(X_j)\mathbb{E}(Y) + \mathbb{E}(X_j)\mathbb{E}(Y)| \\ &= |\mathbb{E}(X_jY) - \mathbb{E}(X_j)\mathbb{E}(Y)| \\ &= |\mathrm{Cov}(X_j, Y)|\,.\end{aligned}$$

(Note that the absolute value function is a continuous function). Therefore, Lemma 1 establishes that $\hat{\tau}_j$ is a consistent estimator of $|\mathrm{Cov}(X_j, Y)|$. □

Lemma 2. It can be shown that $\hat{\sigma}_j$ is a consistent estimator of σ_j and $\hat{\sigma}_Y$ is a consistent estimator of σ_Y.

Proof We begin by noting that, similar unto what was done in rewriting $\hat{\tau}_j$ in Lemma 1, we can rewrite $\hat{\sigma}_j$ as follows:

$$\begin{aligned}\hat{\sigma}_j^2 &= \sum_{k=1}^{K_j}(v_k^{(j)} - \bar{v}^{(j)})^2\hat{p}_k^{(j)} \\ &= \frac{1}{n}\sum_{k=1}^{K_j}(v_k^{(j)} - \bar{v}^{(j)})^2 n_k \\ &= \frac{1}{n}\left((v_1^{(j)} - \bar{X}_j)^2 n_1 + \cdots + (v_{K_j}^{(j)} - \bar{X}_j)^2 n_{K_j}\right) \\ &= \frac{1}{n}\sum_{i=1}^{n}(X_{ij} - \bar{X}_j)^2,\end{aligned} \tag{3}$$

where n_k is the number of times $\{X_{ij} = v_k^{(j)}\}$. Similarly we can obtain that

$$\hat{\sigma}_Y^2 = \frac{1}{n}\sum_{i=1}^{n}(Y_i - \bar{Y})^2. \tag{4}$$

Equations (3) and (4) can both be written using a general notation W as follows. Let $W_1,\ W_2,\ \ldots,\ W_n$ be realizations of a categorical random variable W. Define

$$\hat{\sigma}^2 = \frac{1}{n}\sum_{i=1}^{n}(W_i - \bar{W})^2,$$

where $\bar{W} = \frac{1}{n}\sum W_i$. Let σ^2 denote the variance of W. Define the unbiased sample variance as

$$S^2 = \frac{1}{n-1}\sum_{i=1}^{n}(W_i - \bar{W})^2.$$

We then have

$$\hat{\sigma}^2 = \frac{n-1}{n} S^2. \tag{5}$$

It follows that

$$\mathbb{E}(\hat{\sigma}^2) = \frac{(n-1)}{n}\sigma^2, \quad \mathrm{Var}(\hat{\sigma}^2) = \left(\frac{n-1}{n}\right)^2 \mathrm{Var}(S^2).$$

We can easily show that S^2 is a consistent estimator of σ^2 by the continuous mapping Theorem. We also know that the numeric sequence $\{\frac{n-1}{n}\}$ converges in probability to 1. Applying the Mann-Wald theorem to Equation (5), we obtain that $\hat{\sigma}^2 = \frac{n-1}{n} S^2$ converges in probability to $1 \cdot \sigma^2 = \sigma^2$. This confirms that $\hat{\sigma}^2$ is also a consistent estimator of σ^2. Therefore, the Mann-Wald theorem guarantees that $\hat{\sigma}$ is a consistent estimator of $\sigma = \sqrt{\mathrm{Var}(W)}$. This establishes that $\hat{\sigma}_j$ and $\hat{\sigma}_Y$ are consistent estimators of σ_j and σ_Y. □

Lemma 3. $\hat{\varrho}_j$ is a consistent estimator of ϱ_j.

Proof The definition of $\hat{\varrho}_j$ given in Equation (3) of the main text can be naturally rewritten as

$$\hat{\varrho}_j = \frac{\hat{\tau}_j}{\hat{\sigma}_j \hat{\sigma}_Y}.$$

We can assume without loss of generality that $\hat{\sigma}_j$ and $\hat{\sigma}_Y$ are both positive in line with condition (C1). The Mann-Wald theorem asserts that continuous functions preserve convergence in probability. Therefore, it can be established by Lemmas 1 and 2 that $\hat{\varrho}_j$ is a consistent estimator of the Pearson correlation coefficient. Note that the definition of ϱ_j given in Equation (2) of the main text is the categorical version of the Pearson correlation coefficient. It now completes the proof that $\hat{\varrho}_j$ is the consistent estimator of ϱ_j. □

Appendix B: Proofs of Theorem 1 and Theorem 2

Proof The proofs of these two theorems are accomplished in four steps:

- Step 1: We show that there exists a positive value $\varrho_{\min} > 0$ such that $\varrho_j > \varrho_{\min} > 0$ holds for any $j \in \mathcal{S}_T$ (i.e., Corollary 1). Recall that ϱ_j defined in Equation (2) of the main text is the categorical version of the Pearson correlation coefficient.

  It follows that for $j \in \mathcal{S}_T$,

$$\begin{aligned} \varrho_j = \frac{|\mathrm{Cov}(X_j, Y)|}{\sigma_j \sigma_Y} &\geq \frac{1}{\sigma^2_{\max}} |\mathrm{Cov}(X_j, Y)| \quad \text{by (C1)}, \\ &\geq \frac{\omega_{\min}}{\sigma^2_{\max}} \quad \text{by (C2)}, \\ &> 0. \end{aligned}$$

Define $\varrho_{\min} = \omega_{\min}/(2\sigma^2_{\max})$. Then $\varrho_j > \varrho_{\min} > 0$ for all $j \in \mathcal{S}_{\mathcal{T}}$. This establishes a positive lower bound on ϱ_j for all $j \in \mathcal{S}_{\mathcal{T}}$, and hence completing Step 1 and also Corollary 1.

- Step 2: We now show that $\hat{\varrho}_j$ is a uniformly consistent estimator of ϱ_j for each $1 \leq j \leq p$ (i.e., Corollary 2). From Lemma 3, we know that $\hat{\varrho}_j$ is consistent estimator of ϱ_j. This implies that for any $1 \leq j \leq p$ and any $\varepsilon > 0$, we have

$$\mathbb{P}(|\hat{\varrho}_j - \varrho_j| > \varepsilon) \to 0 \quad \text{as } n \to \infty.$$

Let $J = \text{argmax}_{1\leq j\leq p} |\hat{\varrho}_j - \varrho_j|$. Then, since $J \in \{1, 2, \ldots, p\}$ itself, we indeed know that

$$\mathbb{P}(|\hat{\varrho}_J - \varrho_J| > \varepsilon) \to 0 \quad \text{as } n \to \infty$$

for any $\varepsilon > 0$. In other words, we have that

$$\mathbb{P}\left(\max_{1\leq j\leq p} |\hat{\varrho}_j - \varrho_j| > \varepsilon\right) \to 0 \quad \text{as } n \to \infty$$

for any $\varepsilon > 0$. This shows that $\hat{\varrho}_j$ is a *uniformly* consistent estimator of ϱ_j, thus completing Step 2 and also Corollary 2.

- Step 3: We show that there exists a positive constant $c > 0$ such that

$$\mathbb{P}(\mathcal{S}_T \subseteq \widehat{\mathcal{S}}) \to 1 \text{ as } n \longrightarrow \infty.$$

Note that this is the sure screening property stated in Theorem 1.
Let $c = (2/3)\varrho_{\min}$. Suppose by way of contradiction that this c is insufficient to be able to claim $\mathcal{S}_T \subseteq \widehat{\mathcal{S}}$. This would mean that there exists some $j^* \in \mathcal{S}_T$, yet $j^* \notin \widehat{\mathcal{S}}$. It then follows that we must have (by the definition of $\widehat{\mathcal{S}}$ defined in Equation (4) of the main text)

$$\hat{\varrho}_{j^*} \leq (2/3)\varrho_{\min},$$

while at the same time having (by the conclusion of Corollary 1)

$$\varrho_{j^*} > \varrho_{\min} > (2/3)\varrho_{\min}.$$

From this we can conclude that $|\hat{\varrho}_{j^*} - \varrho_{j^*}| > (1/3)\varrho_{\min}$, which implies that

$$\max_{1\leq j\leq p} |\hat{\varrho}_j - \varrho_j| > |\hat{\varrho}_{j^*} - \varrho_{j^*}| > (1/3)\varrho_{\min}.$$

By the uniform consistency of $\hat{\varrho}_j$ stated in Step 2 when setting $\varepsilon = 1/3\varrho_{\min}$, we have

$$\mathbb{P}(\mathcal{S}_T \not\subseteq \widehat{\mathcal{S}}) \leq \mathbb{P}\left(\max_{1\leq j\leq p} |\hat{\varrho}_j - \varrho_j| > (1/3)\varrho_{\min}\right) \to 0 \quad \text{as } n \to \infty.$$

This is a contradiction to the assumption of non containment above. This yields

$$\mathbb{P}(\mathcal{S}_T \subseteq \widehat{\mathcal{S}}) \to 1 \quad \text{as } n \to \infty,$$

proving Theorem 1.

- Step 4: We show that there exists a positive constant $c > 0$ such that

$$\mathbb{P}(\widehat{\mathcal{S}} \subseteq \mathcal{S}_T) \to 1 \text{ as } n \to \infty.$$

Suppose again by way of contradiction that $\widehat{\mathcal{S}} \not\subseteq \mathcal{S}_T$. Then there is some $j^* \in \widehat{\mathcal{S}}$, yet $j^* \notin \mathcal{S}_T$. This means that

$$\hat{\varrho}_{j^*} > (2/3)\varrho_{\min},$$

while at the same time (by condition (C2)) having

$$\varrho_{j^*} = 0.$$

It now follows that

$$|\hat{\varrho}_{j^*} - \varrho_{j^*}| > (2/3)\varrho_{\min},$$

which implies that $\max_{1\le j\le p} \; |\hat{\varrho}_j - \varrho_j| > (2/3)\varrho_{\min}$ as well.
Set $\varepsilon = (2/3)\varrho_{\min}$. By uniform consistency we have

$$\mathbb{P}(\widehat{\mathcal{S}} \not\subseteq \mathcal{S}_T) \le \mathbb{P}\left(\max_{1\le j\le p} |\hat{\varrho}_j - \varrho_j| > (2/3)\varrho_{\min}\right) \to 0 \quad \text{as } n \to \infty.$$

This is a contradiction to the assumption of non containment above. Hence it in fact follows that

$$\mathbb{P}(\widehat{\mathcal{S}} \subseteq \mathcal{S}_T) \to 1 \quad \text{as } n \to \infty.$$

Combining Step 3 and Step 4 together, we conclude that for $c = (2/3)\varrho_{\min}$, we have $\mathbb{P}(\mathcal{S}_T = \widehat{\mathcal{S}}) \to 1$ as $n \to \infty$, completing the proof of Theorem 2.

□

Appendix C: Additional Results of Real Data Analyses

Author details
[1]Department of Mathematical Sciences, SUNY Binghamton University, Vestal, New York. [2]Idaho National Laboratory, Idaho Falls, Idaho.

References

1. Mann, H.B., Wald, A.: On stochastic limit and order relationships. The Annals of Mathematical Statistics **14**(3), 217–226 (1943)
2. Serfling, R.J.: Approximation Theorems of Mathematical Statistics. Wiley, New York (1980)
3. Casella, G., Berger, R.L.: Statistical Inference. Duxbury advanced series in statistics and decision sciences. Thomson Learning, Ithaca, NY. (2002)

[width=250pt,height=350pt]FinalGene.pdf

Figure 1 Results of the most influential SNPs selected by the ITC-SIS+multiple logistic regression approach. We summarize the estimated ITC score, $\hat{\beta}$ coefficient, p-value, corresponding gene name, Allele type, and detailed position for each of the 53 selected influential SNPs that could locate nearby genes. We confirm many genes that were reported in PCOS literature, direct genes are highlighted in yellow, and the nearby genes are highlighted in green. All other genes without highlighted are new genes that are located by our approach.
